# Performance, safety and efficiency comparison between a 25G 20,000 and a 10,000 cuts per minute vitrectomy: a prospective randomized control study

**DOI:** 10.1186/s40942-024-00613-w

**Published:** 2025-04-14

**Authors:** Shing Chuen Chow, Jeffrey Man Yeung Lo, Mehnaz Quddus, Qing Li, Wai Ching Lam, Nicholas Siu Kay Fung

**Affiliations:** 1 The Li Ka Shing Faculty of Medicine, The University of Hong Kong, Pok Fu Lam, Hong Kong; 2 Queen Mary Hospital & Grantham Hospital, Aberdeen, Hong Kong; 3 Faculty of Medicine, The University of British Columbia, Vancouver, Canada; 4 Hong Kong Santorium & Hospital, Room 301, Level 3 Block B, Cyberport 4 100 Cyberport Road, Happy Valley, Hong Kong

**Keywords:** Vitrectomy, Core duration of vitrectomy, Intraoperative retinal tear, Post-operative retinal tear, Intraoperative complication, Postoperative complication

## Abstract

**Background:**

The aim of this study was to compare the safety and efficacy of 20,000 cuts per minute (cpm) with 10,000 cpm in vitreous cutters.

**Methods:**

This was a prospective, parallel, single masked randomized control trial comparing the 25 gauge 20,000 cpm HYPERVIT Dual Blade from Alcon Laboratories, Fort Worth, TX, USA and 10,000 cpm ULTRAVIT vitrectomy cutter from Alcon Laboratories, Inc, Fort Worth, TX. Standard T-test by SPSS version 27 was used to compare efficiency and safety between two groups.

**Results:**

In total 72 patients were recruited for the study and among them 71 patients completed the study. This study did not show any significant difference between 20,000 cpm probe and 10,000 cpm probe (*p* value = 0.347) for the core vitrectomy duration in all included eyes. The mean of core vitrectomy time was 269.28 s in the 25 gauge 20,000 cpm group and 289.44 s in the 25 gauge 10,000 cpm group. However, by comparing the two systems operated on epiretinal membrane eyes, 20,000 cpm probe had a significantly shorter mean core vitrectomy time than 10,000 cpm group (*P* = 0.03). The majority of all the patients had no intraoperative retinal tear (98.6.8%) and post-operative retinal tear (95.8%). There were no intraoperative iatrogenic breaks, and 3 postoperative retinal tears with rhegmatogenous retinal detachment (RRD) were documented. All the retinal tears belongs to the 20,000 cpm group but no significant difference was found between the two groups in terms of retinal tear and complications.

**Conclusions:**

25-gauge 20,000 cpm Hypervit dual blade showed a faster trend in vitrectomy time although this was not statistically significant in all included eyes. By comparing vitrectomy time operated on epiretinal membrane eyes, a significant shorter time was found in 25-gauge 20,000 cpm. With more efficient and faster vitrectomy systems, the effect of surgeon factor likely plays a larger role. Our study suggest that the two devices may have a similar efficacy and safety. However, further studies may be needed to compare the core vitrectomy time between them after excluding the surgeon factor influence.

## Background

Since the invention of the first vitreous cutter by Robert Machemer, there has been enormous advancement in the field of vitreoretinal surgery [1, 2]. Different vitrectomy systems have been developed, including the historical gold standard 20 gauge vitrectomy, followed by the 25 gauge, and now 27 gauge [3–6]. Small gauge incisions provided multiple advantages in terms of wound construction, reduction of operation time, postoperative inflammation and complication rate [7]. The 25 gauge vitrectomy system continued to evolve after it was introduced in 2002 in terms of cutting speed, cutting port surface area enlargement and the internal shaft diameter [3, 6, 8–11].

The increase in cutting rate tends to provide a shorter vitrectomy time and a better safety profile [8, 11–16]. Theoretically, the reduction of vitrectomy time is due to improvement in vitreous removal efficiency. This is achieved by cutting vitreous into smaller pieces, reducing viscosity and resistance of vitreous flow. Improvement in safety is due to the reduction of uncut vitreous entering the port, thus lower the traction generated on the adjacent retina. Ultimately, this may lessen the chance of an iatrogenic retinal break [3, 14–16]. Various clinical studies have investigated the safety and efficiency profile of different cutter speeds in 25-gauge vitrectomy system [8, 12, 13]. Rizzo et al. increased the cutting rate from 1,500 cpm to 5,000 cpm and demonstrated a better efficiency and safety profile [13]. The increase in cutting rate from 5,000 cpm to 7,500 cpm showed significant reduction in vitrectomy time but no difference in safety profile [13]. No significant difference in vitrectomy time and complication rate was demonstrated in a recently published study by comparing 10,000 cpm and 5,000 cpm, despite shorter mean vitrectomy time was shown [11]. The mild variation may be due to other contributing factors such as aspiration rate, cutter speed, pot size, duty cycle, ocular diseases and surgeon preferences [11, 17].

Duty cycle refers to the percentage of time probe port remains open. In old designs, it was adversely affected by the ultrahigh speed cutting [8]. Spring return mechanism was a system used in the past. Increasing the cutting rate will result in lowering the duty cycle in sprint return mechanism vitreous cutter [11]. A newer mechanism, dual pneumatic drive technology was implemented a few years ago and allows a high cut rate without compromising the duty cycle [11, 18]. It provided vitrectomy system with a chance to achieve ultrahigh speed, up to 10,000–15,000 cpm [18]. Recently, a new dual blade design was introduced and the newly launched HYPERVIT Dual Blade is available in 25 and 27 gauge size. Due to the dual blade and additional port design, it is able to achieve a nearly continuous duty cycle and cuts twice per cycle [16]. Therefore, it is possible to cut at 20,000 cut per minute, twice the speed of Advanced ULTRAVIT Probe. It is also equipped with a bevelled tip, which allow a closer distance between the cutting port and retina when compared to standard flat-tipped probes.

Various non clinical studies had compared dual blade vitrectomy versus single blade in terms of flow dynamics [16, 18–22] and demonstrated a better fluidic performance. A retrospective study from Japan compared 27 gauge 20,000 cpm and 10,000 cpm, showed a significant reduction in vitrectomy time. To our knowledge, there has yet to be a randomised control study comparing the efficiency and safety of a 25 gauge 20,000 cuts/min and a 25 gauge 10,000 cuts/min vitrectomy.

## Methods

This was a prospective, parallel, single masked randomized control trial aiming to compare the 25 gauge 20,000 cpm HYPERVIT Dual Blade from Alcon Laboratories, Fort Worth, TX, USA and 10,000 cpm ULTRAVIT vitrectomy cutter from Alcon Laboratories, Inc, Fort Worth, TX. The study took place in Grantham Hospital and the Department of Ophthalmology in Queen Mary Hospital after gaining approval from the governing research and ethics committee (Institutional Review Board of the University of Hong Kong/Hospital Authority Hong Kong West Cluster (“HKU/HA HKWC IRB”)) (UW21-384). This study adhered to the tenets of the Declaration of Helsinki. The study was also registered with clinicaltrials. Gov (NCT05710458) and the Hong Kong West Cluster Ethics Board (UW21-384).

Patients over age of 18 requiring vitrectomy between July 2021 and July 2022 were recruited for this study. Surgical indications include epiretinal membrane, macular hole, vitreous haemorrhage or primary retinal detachment. A computer-generated randomization list was used for randomization of participants. Exclusion criteria for patients include eyes with ocular comorbidities affecting surgical view (e.g. corneal opacities), eyes with history of previous vitrectomy/scleral buckle surgery, trauma or requiring silicone oil, patient unable to give proper consent, undergoing repeated retinal detachment surgery or surgery for tractional diabetic retinopathy.

### Study procedures

All surgeries were performed by three qualified vitreoretinal surgeons. Prior to surgery, baseline ophthalmic examination including best corrected visual acuity (BCVA), intraocular pressure, and past medical history were assessed. Core vitrectomy duration and any complications were recorded for each surgery. Core vitrectomy duration was recorded as the duration the vitrector was activated by the Alcon Constellation^®^ Vision system. Patients had follow-up for 3 months postoperatively to look for any postoperative complications. The primary outcome of the study was the duration of core vitrectomy while the secondary outcome was the intraoperative and postoperative complications. Core vitrectomy time was also compared between the two groups performed by each surgeon. Comparison of eyes with preoperative diagnosis of epiretinal membrane between Group A and B was performed. Complications and other incidents that was observed by study personnel or reported by the participant were recorded in the subject data sheet. Complications were followed to an adequate resolution.

### Statistical methods

We used SPSS version 27 for all our data analysis. Standard t-test was performed to compare the result between 2 groups: 20,000 cpm and 10,000 cpm. A descriptive summary is presented for all study endpoints. All continuous variables are presented with the following summary statistics: mean, standard deviation, median, min, max. For the comparison of the two groups, an independent T-test was performed. Categorical variables are summarized by count and percentage. Comparison between groups was performed by Fisher’s exact test for categorical variables.

### Sample size justification

There is a comparative study looking at high speed vitrectomy cutters for the sample size calculation. Cesare M et al. 2015 reported the mean ± standard deviation of core vitrectomy time was 161.32 ± 39.10 s in the 25 gauge 7,500 cpm Group and 184.10 ± 41.69 s in the 25 gauge 5,000 cpm Standard Group [13]. The observed difference in mean core vitrectomy duration between subjects treated with 7,500 cpm probes and those in the Standard Group was 22 s. With the assumption, the higher cutting speed (20,000 cpm) will take at least 25 s less time in doing the core vitrectomy than the 10,000 cpm group, at a one-sided 0.05 significant level, with a common standard deviation of 40.4 s, 24 patients at each group will have 80% power to achieve the study primary objective.

## Results

A Total of 72 patients (72 eyes) were enrolled in the study (36 Group A: 20,000 cpm HYPERVIT Dual Blade and 36 Group B: 10,000 cpm ULTRAVIT vitrectomy cutter) (Table 1). The mean age for Group A and Group B was 68.9 and 70.3 years old respectively. Group A had 50% male and Group B had 61% male. Diagnoses included were 46 epiretinal membrane, 15 macular hole, 9 vitreous haemorrhage and 2 retinal detachments. The majority of the patients underwent vitrectomy for ERM (69.4% for Group A and 58.3% for Group B) followed by full thickness macular hole (22.2% for Group A and 19.4% for Group B). No significant difference was found between the two groups at baseline in terms of age and IOP. There was a significant difference between baseline BCVA (*p*-value = 0.044) and 3 months postoperative follow-up BCVA (*p*-value = 0.034) between the two groups (Table 2).

**Table 1:** Baseline characteristics of all included patients

Characteristics	Group A (***n*** = 36)	Group B (***n*** = 36)
Age (mean ± SD)	68.78 ± 7.27	70.28 ± 11.08
Gender (M: F)	18:18	22:14
Eyes (R: L)	14:22	19:17
**Preoperative Ocular Diagnosis**:		
Epiretinal Membrane, n(%)	25(69.4%)	21(58.3%)
Macular Hole, n(%)	8(22.2%)	7(19.4%)
Retinal Detachment, n(%)	1(2.8%)	1(2.8%)
Vitreous Haemorrhage, n(%)	2(5.6%)	7(19.4%)

**Table 2:** IOP, BCVA and CorVit Duration of all included patients

	Group A						Group B						***P*** value (t test)
	***N***	Mean	Median	Std. Deviation	Min	Max	***N***	Mean	Median	St. Deviation	Min	Max	
CorVit Duration	36	269.28	247.00	97.370	118	475	36	289.44	279.00	82.893	151	536	0.347
BCVA Baseline	36	0.289	0.300	0.202	0.005	0.700	36	0.194	0.175	0.193	0.005	0.700	0.044*
IOP Baseline	19	17.079	17.000	2.780	12.0	23.0	21	15.605	16.000	2.663	10.0	20.0	0.095
BCVA after 3 months	36	0.439	0.450	0.256	0.010	1.00	35	0.304	0.200	0.269	0.005	1.00	0.034*
IOP after 3 months	32	17.84	18.00	3.323	11	24	27	16.67	17.00	2.760	11	21	0.149

**Fig. 1:**
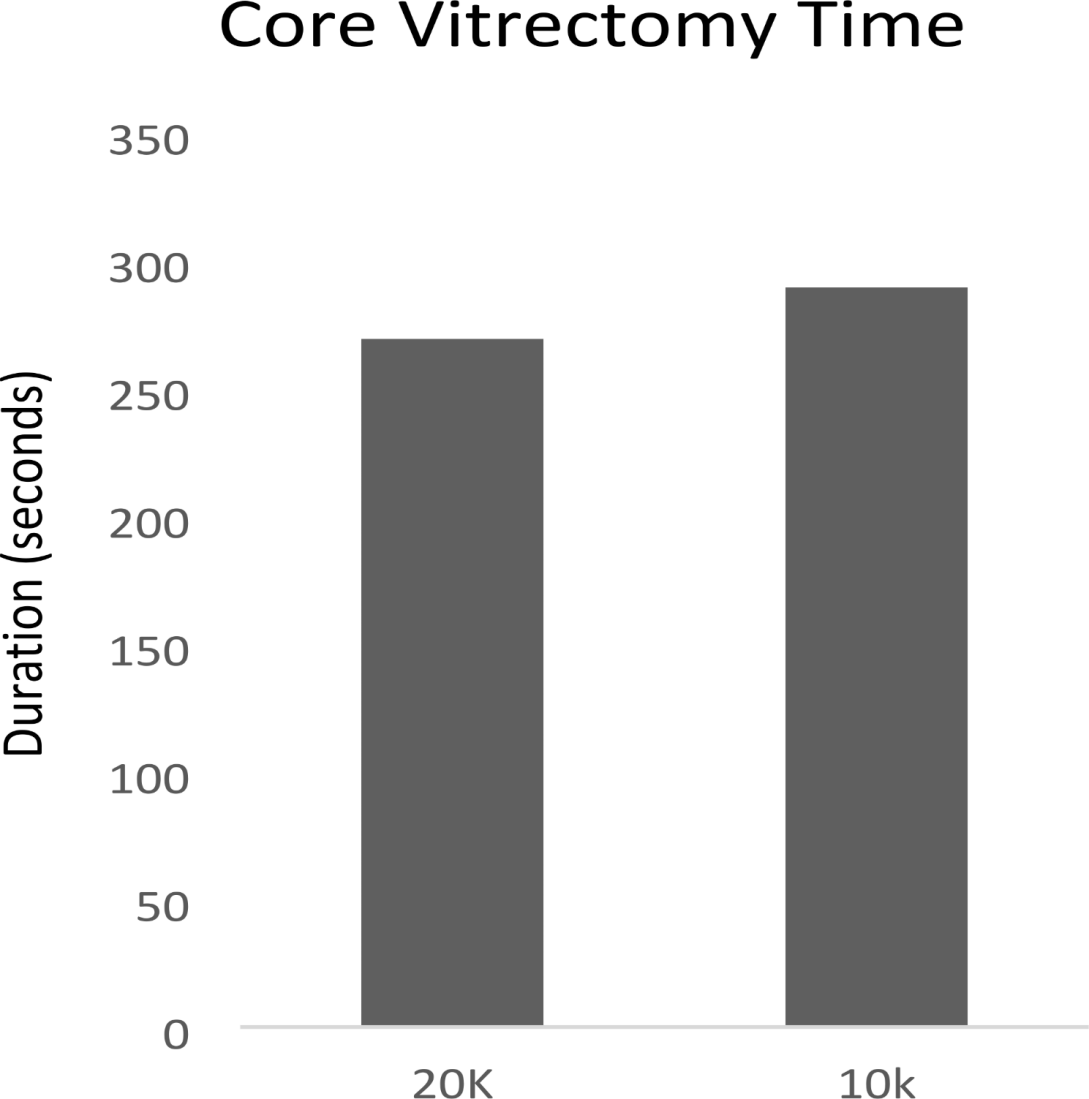
Comparison of vitrectomy time in seconds between 20 K and 10 K in all included patients

The mean core vitrectomy time for Group A and B of all included patients was 269.28 ± 97.37 s and 289.44 ± 82.89 s respectively (Fig. 1), (Table 2). The mean difference in core vitrectomy duration between the two groups was 20.16 s and the 95% confidence interval was 2.9 to -5.9. Core vitrectomy duration for 20,000 cpm probe was not significantly shorter than 10,000 cpm probe (*P* = 0.347). Subgroup analysis of eyes performed by different surgeons also showed no significance difference between 20,000 cpm HYPERVIT Dual Blade and 10,000 com ULTRAVIT vitrectomy cutter. A total of three surgeons participated in our study. A total of 19 eyes were operated by one of our surgeons, (11 eyes by 20,000 cpm HYPERVIT Dual Blade and 8 eyes by 10,000 cpm ULTRAVIT vitrectomy cutter). The mean core vitrectomy time was 207.00 ± 87.90 vs. 233.00 ± 74.89 respectively (*P* = 0.508). Another surgeon had operated on 22 eyes (11 eyes in each group). No significance difference was found between the two groups (20,000 cpm HYPERVIT Dual Blade: 327.09 ± 109.72 vs. 10,000 cpm ULTRAVIT vitrectomy cutter: 356.73 ± 90.62, *P* = 0.498). Similar results were also demonstrated in eyes operated by our last surgeon. The mean core vitrectomy time was 272.79 ± 64.81 in 14 eyes from the 20,000 cpm HYPERVIT Dual Blade group and 272.47 ± 49.33 in 17 eyes from 10,000 cpm ULTRAVIT vitrectomy cutter group (*P* = 0.99).

Among the 46 eyes with epiretinal membrane, 25 of them belongs to group A and 21 of them eyes belongs to group B (Table 3). The mean core vitrectomy duration in group A epiretinal membrane patients was 241.00 ± 79.76 s while in group B epiretinal membrane patients was 291.19 ± 72.16 (*P* = 0.03). For the 15 eyes with macula hole, no significant difference was found when comparing Group A and Group B (*P* = 0.22, 8 vs. 7 eyes). There was no significant difference found when comparing group A and B among patient with vitreous haemorrhage (*P* = 0.34, 2 vs. 7 eyes). While for RD, each group only had 1 eye included and the mean of core vitrectomy time was 361s in group A and 536 s in group B.

**Table 3:** IOP, BCVA, CorVit Duration of eyes with epiretinal membrane

	Group A						Group B						***P*** value (t test)
	***N***	Mean	Median	Std. Deviation	Min	Max	***N***	Mean	Median	St. Deviation	Min	Max	
CorVit Duration	25	241.00	239.00	79.76	118	399	21	291.19	281.00	72.16	152	432	0.03
BCVA Baseline	25	0.356	0.3	0.188	0.05	0.7	21	0.273	0.3	0.165	0.01	0.7	0.121
IOP Baseline	12	16.6	17.0	2.24	12	20	11	15.6	16.0	2.78	10.7	20	0.350
BCVA after 3 months	25	0.47	0.5	0.244	0.1	1.0	21	0.37	0.3	0.24	0.1	1.0	0.178
IOP after 3 months	23	18.74	19	2.97	12	24	14	16.64	17	3.25	11	21	0.06

Subgroup analysis by comparing the two vitrectomy systems by individual surgeons was also done in epiretinal membrane eyes. One of the surgeons demonstrated a significant reduction in core vitrectomy time when comparing eyes operated on by 20,000 cpm HYPERVIT Dual Blade and 10,000 cpm ULTRAVIT vitrectomy cutter (146 ± 32.63 s vs. 240.00 ± 77.31 s, 6 eyes vs. 3 eyes) (*P* = 0.032). However, for the other two surgeons, neither showed a significant difference in core vitrectomy time. 14 eyes were operated by surgeon X, the mean core vitrectomy time was 273.25 ± 70.98 s in 20,000 cpm HYPERVIT Dual Blade group and 351.00 ± 73.53 s in 10,000 cpm ULTRAVIT vitrectomy cutter group (8 vs. 6 eyes, *P* = 0.069). Moreover, the core vitrectomy time of surgeon Y was 269.36 ± 63.85 by 20,000 cpm HYPERVIT Dual Blade and 274.08 ± 54.22 by 10,000 cpm ULTRAVIT vitrectomy cutter (11 vs. 12 eyes, *P* = 0.85).

There was no significant difference between the two groups irrespective of the intraoperative and postoperative complications (Table 4). There were no intraoperative iatrogenic breaks. There was 1 case of retinal breaks that was discovered during internal search. There were 3 cases of 3 postoperative retinal tear with RRD in group (A) No retinal tear incidents were recorded for group (B) Retinal tear found during the procedure were treated with endolaser. Both intraoperative and postoperative retinal tear were not significantly related irrespective of the vitrectomy probes.

**Table 4:** Complication rate of all included patients

	Group 1	Group 2	Percentage		Exact. Sig. (2-sided) (Fisher’s exact test)
			Group 1	Group 2	
IntraOp_Retinal Tear	1	0	2.8%	0%	0.314
PostOp_Retinal Tear	3	0	8.3%	0%	0.077

## Discussion

The new dual blade design enables the vitrectomy system to achieve a nearly continuous duty cycle and results in continuous aspiration and flow of vitreous. Duty cycle is one of the main factors that affect the flow rate of vitreous humor [8]. The design of cutting twice in each cycle, further increases the number of cpm of the device. Apart from the recently launched 20,000 cpm HYPERVIT Dual Blade that was used in our study, other vitrectomy systems from different manufacturers had also implemented the double blade design. For example, the UNO Colorline MACH2 vitreous cutter was able to achieve 12,000 cpm, the DORC TDC (Two Dimensional cutting) could achieve up to 16,000 cpm and the Bi-Blade could increase up to 15,000 cpm. In previous non-clinical studies, better fluidics and flow rate were demonstrated in dual blade design than single blade design vitrectomy systems [16, 18, 20–22]. One of the non-clinical studies, Inoue et al. showed a significant increase in aspiration rate by a 25 guage dual blade cutter at 20,000 cpm when compared with single blade cutters at 10,000 cpm, by using same device with our study. One of the clinical studies, Pavlidis has demonstrated a faster core vitrectomy by using either a 25 or 27 guage DORC TDC when compared with single bladed standard vitrectome [23]. A retrospective study from Japan also demonstrated similar results [24]. High efficiency of vitreous removal was demonstrated in eyes using 27 gauge vitrectomy 20k cpm versus 10k cpm, same brand used in our study, but different gauge sized vitrectomy cutter. Interestingly, most of the findings of our study had demonstrated no significant difference in core vitrectomy duration between 25 gauge 20,000 cpm HYPERVIT Dual Blade and 10,000 cpm ULTRAVIT vitrectomy cutter, despite Group A showed a shorter mean core vitrectomy time than B and surgeons participated in our study had subjective feeling of better stability and increase in efficacy in HYPERVIT Dual Blade.

Apart from the performance of the vitrectomy system, surgeon preference and performance may also affect the vitrectomy time. With more efficient and powerful vitrectomy systems, the surgeon becomes an important variable that can influence the surgical outcome, as much so as the vitrectomy system design [11]. In our study, measurement of the core vitrectomy time was recorded by the duration the vitrector was activated. Surgical techniques vary and thus the timing of activation of the cutter varies as well. For example, some surgeons may activate the vitrector despite actively engaging with the vitreous. In order to eliminate this influence, our study had tried to perform subgroup analysis of Group A vs. Group B performed by the same surgeon.

Practice of surgeon in performing core vitrectomy and peripheral vitrectomy simultaneously or separately in retinal detachment / macular hole patients may also affect the core vitrectomy time. With regard to this, our study had compared group A and B among patients with only epiretinal membrane. The reason is that limited vitrectomies instead of complete vitrectomies were performed in epiretinal membrane patients [25]. It was proven to be a time-efficient and effective procedure to perform limited vitrectomy in epiretinal membrane patients. Epretinal membrane eyes operated by the new 20,000 cpm HYPERVIT Dual Blade had a significant shorter mean time for core vitrectomy than the older system. However, an important limitation was that after extracting eyes with epiretinal membrane, the number eyes was not able to reach 24 at each group, which was the suggested number to achieve 80% power in C Mariotti et al. [13]. Furthermore, when comparing group A and group B epiretinal membrane eyes by the same surgeon, only one surgeon had a significant reduction in core vitrectomy time when using 25 gauge 20,000 cpm HYPERVIT Dual blade. Therefore, further studies may be needed to investigate difference between 25 gauge 20,000 cpm HYPERVIT Dual Blade and 10,000 cpm ULTRAVIT vitrectomy cutter in core vitrectomy time after excluding the surgeon factor.

One of the most important factors to be studied when considering a novel device is safety. The main benefit of higher cutting speed is it reduces the traction on retina by fragmenting vitreous into smaller pieces, preventing the uncut vitreous from going through the port. It intercepts the flow and attains “Port-based flow-limiting” vitrectomy. With a reduced pulse flow (volume per open-close cycle), this achieves fluidic stability and reduces the motion of detached retina, creating less pulsatile traction to the attached retina [12]. Despite suture-less vitrectomy being a safe procedure, there are known complications such as retinal tear, hypotony, vitreous hemorrhage, retinal detachment, and endophthalmitis [26–28]. Several studies showed a range from 2 to 14% of cases of a retinal break due to vitrectomy [12, 29, 30]. In our study we have 3 cases of postoperative retinal tear with RRD in group A. No statistically significant differences between the two groups was demonstrated (intraoperative retinal tear *P* = 0.31, postoperative retinal tear *P* = 0.077). Among our three included surgeons, one of them had no intraoperative or postoperative complications among all the cases. One surgeon had a case of postoperative retinal tear with RRD and the other remaining surgeon had two cases of postoperative retinal tear complicated by RRD.

The first case of postoperative retinal break with RRD was noted on postoperative day 11 after macular hole surgery. The patient had a history of macular hole with PPV done in the fellow eye, that was also complicated by retinal detachment postoperatively requiring PPV and silicone oil insertion. The patient was known to have abnormal vitreous. She developed a superonasal localized RRD and was successfully treated with pneumatic retinopexy with intravitreal C3F8 and enjoys good vision. The second case of postoperative retinal detachment was noted on postoperative day 6 after epiretinal membrane peeling. Superior retinal breaks with a localized RRD were noted and successfully treated with PPV and intravitreal C3F8. The third and final case of postoperative RRD was noted on postoperative day 10. The patient had vitreomacular traction with epiretinal membrane, and membrane peeling with intravitreal SF6 injection was performed. On day 10 post-operatively, choroidal effusion and inferior bullous RRD was noted. He was treated with PPV and C3F8 but developed redetachment, requiring silicone oil insertion.

Surgeon performance and surgical outcome may appear relatively easy to assess based on complication rate, however, complexity of cases and nuances of pathologies allowed for. The first particular case had a history of abnormal vitreous, bilateral macular holes, and a history of RRD following surgery in the fellow eye.

Another key factor is the relation between surgical experience and outcome. Evidence has shown that higher volumes of surgical work and activity correlate with better outcomes [31, 32]. Of the 3 surgeons included in the study, 2 possess over 15 years of vitreoretinal experience and combined for only 1 postoperative complication. Although well trained and highly motivated, errors in surgery do occur and surgeons are fallible, especially when equipped with less experience.

Our study confirms that the 25 gauge 20,000 cpm Hypervit dual blade has similar efficacy and safety as compared to the 10,000 cpm Ultravit vitrectomy cutter. However, limitations were present. Bias may have been induced as this was a single-masked randomized trial, and surgeons were aware of the group and the vitrectomy probe they used. Other limitations include the heterogeneity of ocular pathology and surgeons. Moreover, despite having three qualified vitreoretinal surgeons, surgical experience and preference varied. There may also be a learning curve to the new 20,000 cpm Hypervit probe as surgical settings may also require fine-tuning. To the best of our knowledge to date, there is no prospective randomized controlled trial to show the non-inferiority of intraoperative and postoperative complication, and the comparison of core duration of vitrectomy between 25 gauge 20,000 cpm probe and 10,000 cpm probe.

## Conclusion

25-gauge 20,000 cpm Hypervit dual blade and the 10,000 cpm Ultravit cutters are both similarly safe and efficient options for vitrectomy in various vitreoretinal indications. 25-gauge 20,000 cpm may have a higher efficiency when operated on epiretinal membrane eyes. The limiting factor of vitrectomy time are likely related to surgeon preference and technique or case by case complexity rather than the cutter design. Our study suggests that further studies may be needed to find significant differences in core vitrectomy time between the two cutters after excluding the surgeon factor influence.

## Data Availability

Data available by request to corresponding author up to 3 years after publication.

## Abbreviations

cpm: Cuts per minute
RRD: Rhegmatogenous retinal detachment
BCVA: Best corrected visual acuity
